# Segmenting and classifying lung diseases with M-Segnet and Hybrid Squeezenet-CNN architecture on CT images

**DOI:** 10.1371/journal.pone.0302507

**Published:** 2024-05-16

**Authors:** Syed Mohammed Shafi, Sathiya Kumar Chinnappan

**Affiliations:** School of Computer Science and Engineering Vellore Institute of Technology, Vellore, India; Soochow University, CHINA

## Abstract

Diagnosing lung diseases accurately and promptly is essential for effectively managing this significant public health challenge on a global scale. This paper introduces a new framework called Modified Segnet-based Lung Disease Segmentation and Severity Classification (MSLDSSC). The MSLDSSC model comprises four phases: "preprocessing, segmentation, feature extraction, and classification." Initially, the input image undergoes preprocessing using an improved Wiener filter technique. This technique estimates the power spectral density of the noisy and original images and computes the SNR assisted by PSNR to evaluate image quality. Next, the preprocessed image undergoes Segmentation to identify and separate the RoI from the background objects in the lung image. We employ a Modified Segnet mechanism that utilizes a proposed hard tanh-Softplus activation function for effective Segmentation. Following Segmentation, features such as MLDN, entropy with MRELBP, shape features, and deep features are extracted. Following the feature extraction phase, the retrieved feature set is input into a hybrid severity classification model. This hybrid model comprises two classifiers: SDPA-Squeezenet and DCNN. These classifiers train on the retrieved feature set and effectively classify the severity level of lung diseases.

## 1. Introduction

Image segmentation is the process of partitioning an image into non-overlapping, distinct regions, and it represents a fundamental task in CVPR. This technique finds extensive use in various applications, including the detection and diagnosis of pulmonary diseases [1–3]. In medical imaging, particularly in CT, accurate Segmentation plays a crucial role in identifying respiratory pathologies such as pulmonary fibrosis, lung carcinoma, and pleural effusion. The segmentations originate from volumetric lung representations composed of sequential 2D images called CT slices. Diseases can be effectively identified and measured by meticulously analyzing the organ’s anatomy in these scans and exploring subtle features within CT images. DL techniques offer a promising avenue for this task, allowing for the extraction of intricate and interconnected features from the images, thereby enhancing the accuracy of disease detection and diagnosis [4–6].

Lung segmentation is a crucial initial step in the medical image analysis acquired for assessing lung diseases. Researchers have developed various methods for lung segmentation, broadly categorized into hand-crafted feature-based approaches and DL-based techniques [7]. Unlike hand-crafted methods like region growing, active contour models, and morphological-based models, DNN-based methods can automatically learn representative features without requiring manual empirical parameter adjustments [8, 9]. Traditional hand-crafted feature-based segmentation schemes often require manual parameter tuning. They are designed for specific imaging modalities, applications, and datasets, making generalizing across distinct datasets or CT images challenging. Additionally, these methods often involve user intervention to adjust features and parameters interactively. In contrast, deep learning-based approaches typically require fewer data-specific hyperparameters and tend to outperform hand-crafted methods [10–12].

Recently, DL has emerged as a potent tool for CT segmentation, providing a hierarchical and scalable representation of lung structures. While DL-based approaches have demonstrated effectiveness, many lack user intervention capabilities, disregarding the versatility and autonomy offered through interactive segmentation schemes. Some researchers have attempted to combine DL with seeded image segmentation to reduce the burden of user marking. Yet, these methods were not tailored for chest CT images and still relied heavily on user involvement to achieve satisfactory results [13–15]. Moreover, creating seed maps for numerous CT scans is tedious, labour-intensive, and requires specialized medical expertise to mark specific lesions and lung tissues, such as those caused by COVID-19. Additionally, DL-based segmentation schemes often necessitate a considerable volume of CT data for model training, making the training process highly dependent and time-consuming on specific datasets and ground-truth labelling. This dependency can reduce accuracy, mainly when dealing with CT scans containing multiple lesions of varying sizes and shapes caused by COVID-19 [16, 17]. Deep learning techniques have shown promise in medical image analysis. Leveraging these advancements can lead to more accurate and efficient disease classification. However, DL models require large annotated datasets for training, but obtaining high-quality labeled lung tumor data is challenging due to the scarcity of such data. This impacts limited data which can lead to overfitting or suboptimal performance. Deep convolutional neural networks (CNNs) used in DL require substantial computational resources for training and inference. This impacts real-time segmentation during clinical workflows which is challenging in existing works [18, 19]. To overcome these drawbacks, this paper proposes a novel MSLDSSC framework for Lung disease segmentation and classification. Here, both M-Segnet and Hybrid Squeezenet-CNN architectures are designed to have significantly fewer parameters compared to traditional DL models. This reduction in model complexity leads to faster training, efficient memory usage, and easier deployment on resource-constrained devices. M-Segnet and Hybrid Squeezenet-CNN architectures are designed for efficiency without compromising accuracy. These architectures address computational constraints and allow real-world deployment.

The main contribution of this work is as follows:

- Introduces a new framework called Modified Segnet-based Lung Disease Segmentation and Severity Classification (MSLDSSC) in which improved wiener filtering is proposed for preprocessing the input image. Here, the power spectral density is estimated; following this, PSNR and SNR are computed to evaluate the image quality.
- Adopts a new Modified Segnet model for the segmentation procedure. The Mixed stochastic polling and a new activation function are applied in this model.
- Proposing a Modified LDN for the feature extraction process. For efficient extraction, a new formulation is introduced in the Gaussian mask that maximizes the effectiveness of edge detection. This feature extracts entropy with MRELBP, shape, and deep features.
- Contributes a hybrid severity classification model that comprises SDPA-SqueezeNet and DCNN model. In the SDPA-SqueezeNet model, a scale dot product attention mechanism with improved loss function is determined to classify lung disease effectively.

The remaining paper is organized as follows: Section 2 explains the literary works of extant approaches. The MSLDSSC frameworks’ methodology is elucidated in section 3, the analysis and demonstration of the proposed work are given in section 4, and the conclusion is summarized in section 5.

## 2. Literature review

In 2022, Aldimir et al. [20] developed a semi-automatic framework for segmenting lung CT images of COVID-19 patients by combining deep contour learning with seeded Segmentation. Their DL-driven method utilized label diffusion maps against integrating a network of contour detection with a label propagation model, allowing seed spreading over CT images for Segmentation. The model learned seed diffusion from marked CT scans, enabling iterative and unsupervised Segmentation of multiple CT slices. The effectiveness of this approach was confirmed through quantitative and qualitative assessments, comparing it with user-guided segmentation methods across eight CT datasets with diverse COVID-19 lesions.

In 2023, Mohamed et al. [21] proposed integrating SENets within the IoMT framework to automate the Segmentation of COVID-19 infections in lung CT images. This integration incorporated SE blocks directly into DRN to create Seresnets based on LinkNet and U-Net models. Moreover, the research presented an advanced tool for radiologists to automatically segment COVID-19-infected areas using CT scans, potentially enhancing medical diagnosis routines for positive COVID-19 patients.

In 2022, Guowei et al. [22] proposed a novel lung parenchyma segmentation method is introduced, integrating a two-dimensional reciprocal cross-entropy multi-threshold approach with an enhanced firefly algorithm. An optimal threshold method was initially applied for lung segmentation, enabling dynamic adjustments in segmentation thresholds based on detailed anatomical features like ground-glass opacity, lung lobes, bronchi, and trachea. Moreover, this approach significantly improved lung parenchyma segmentation’s efficacy and enhanced CT image contrast clarity. Experimental results highlighted its effectiveness, especially in cases related to COVID-19, showcasing ideal segmentation outcomes with high robustness and accuracy.

In 2023, Yuan et al. [23] examined the SuperMini-Seg as a lightweight segmentation network that featured the innovative TPCM, which combined transformer and convolution operations within a single module. This network employed a double-branch parallel structure for downsampling images and incorporated a gated attention mechanism between the two branches. Despite its scalability, SuperMini-Seg-V2, containing over 70K parameters, has achieved segmentation accuracy close to that of state-of-the-art methods. Its high computational efficiency made it suitable for practical deployment.

In 2022, Chaodong et al. [24] designed a novel GFNet for COVID-19 lung infections, utilizing VGG16 as the backbone. The network incorporated an Eg module that fused features at every layer. At first, the features were retrieved using a reverse attention module, combined with Eg. This approach enabled all layers to widely extract boundary information, addressing the issues of recognizing fuzzy infected regions. Moreover, the multi-layer output features were fused to segment infected areas accurately and automatically. Comparative analysis was conducted against extant medical segmentation networks such as the latest model Inf-Net, UNet, UNet++, and few-shot learning methods.

In 2022, Jianning et al. [25] proposed a MID-UNet was developed to segment COVID-19 infections in lung CT images. The network utilized various inputs, including an image enhanced by adaptive histogram equalization, the original CT image, an image filtered through a non-local means filter, and a blurry feature map. DCBs were employed to refine features extracted from shortcut connections before transferring them to the de-convolution parts. Moreover, the experimental outcomes on the COVID-19-CT-Seg dataset demonstrated that the proposed MID-UNet surpassed traditional schemes in segmenting COVID-19 infections from CT images.

In 2020, Jiaxing et al. [26] presented a novel lung segmentation approach based on GAN, termed LGAN. LGAN was evaluated on datasets from LIDC-IDRI using two measures: shape similarity and segmentation quality. Moreover, they compared LGAN with contemporary, traditional methods. Furthermore, the experimental results demonstrated the potential of LGAN as an effective tool for automatic lung segmentation owing to its streamlined procedure, enhanced performance, and efficiency.

In 2021, Jinzhu et al. [27] developed an efficient 3D U-Net, improved with ResNet architecture and a dual-pathway deep supervision mechanism, was developed to enhance the network’s capacity to learn extensive representations of lung tumors from both global and local viewpoints. Experimental results demonstrated that the proposed 3D MSDS-UNet outperformed existing segmentation schemes, showing significant advancements in segmenting tumors across all sizes, especially displaying notable enhancements in accurately segmenting small tumors.

In 2023, Shimpy Goyal and Rajiv Singh [28] proposed a framework for predicting lung diseases from chest X-ray images encompassing several stages: “dataset acquisition, image quality enhancement, adaptive RoI estimation, feature extraction, and disease anticipation.” Moreover, the two publicly available chest X-ray datasets were utilized, and image quality was improved using histogram equalization and median filtering due to inherent degradation. Also, a modified region-growing mechanism ensured the integration of dynamic region selection, accurate RoI extraction, and morphological operations. Classification tasks utilize soft computing methods such as ANN, ensemble classifiers, KNN, SVM, and DL classifiers. Experimental results underscore the framework’s efficiency and robustness, outperforming existing methods.

In 2021, Min Hong et al. [29] presented a novel approach for classifying lung disease images using CNNs. The datasets utilized included the NIH dataset, divided into Normal, Pneumothorax classes, and Pneumonia. The Multi-GAP structure was also employed to maximize feature utilization from each layer. Experimental results on the NIH dataset demonstrated that the proposed method achieved the highest performance among tested models. This approach showcased promising advancements in accurately classifying lung disease images, offering potential medical diagnosis and treatment planning benefits.

### 2.1 Problem statement

The reviews based on lung segmentation using CT images are shown in Table 1. Initially, the DL-driven approach was introduced in [20], which provides Lower computation time, higher accuracy, and minimal loss. However, the adopted framework must simultaneously address segmentation and disease classification tasks. The SENets were developed in [21] that offer a structure similarity index, higher Dice score, enhanced alignment measure, and low mean absolute error; nevertheless, the chosen approach necessitated the inclusion of additional datasets from various medical imaging modalities. The improved firefly algorithm was employed in [22], which offers less running time, good accuracy, and sensitivity; however, further investigation is required to explore variations in segmentation accuracy across different CT images. Also, the SuperMini-Seg model was employed in [23], providing high efficiency and segmentation accuracy. However, different feature fusion methods for transformers and CNNs should be experimented with to enhance the network’s performance. Likewise, the GFNet model was presented in [24], which offers precision, sensitivity, specificity, and a higher Dice similarity coefficient. However, there is a need to apply the GFNet framework to additional medical image segmentation tasks, including cellular structures and colonoscopic polyps, and image segmentation in other domains. In addition, the MID-UNet model was deployed in [25], ensuring higher sensitivity, specificity, and a higher dice coefficient.

**Table 1:** Features and challenges on existing systems.

Author [Citation]	Methodology	Features	Challenges
Aldimir et al. [20]	DL-driven approach	Lower computation time, higher accuracy, and minimal loss were achieved.	The framework adopted needs to address both segmentation and disease classification tasks simultaneously.
Mohamed et al. [21]	SENets	The structure similarity index, higher Dice score, enhanced alignment measure, and low mean absolute error were observed.	The chosen approach necessitated the inclusion of additional datasets from various medical imaging modalities.
Guowei et al. [22]	Improved firefly algorithm	Less running time, good accuracy, and sensitivity were achieved.	Further investigation is required to explore variations in segmentation accuracy across different CT images.
Yuan et al. [23]	SuperMini-Seg model	High efficiency and high segmentation accuracy were attained.	Different feature fusion methods for transformers and CNNs should be experimented with to enhance the network’s performance.
Chaodong et al. [24]	GFNet model	Precision, sensitivity, specificity, and higher Dice similarity coefficient were observed.	It is necessary to apply the GFNet framework to additional medical image segmentation tasks, including cellular structures and colonoscopic polyps, and image segmentation in other domains.
Jianning et al [25]	MID-UNet model	Sensitivity, specificity, and a higher dice coefficient were noted.	Clinicians must differentiate between various infected areas to assess the condition accurately.
Jiaxing et al [26]	LGAN model	Improved robustness and better Dice score were observed.	There is a need to expand the proposed framework to include Segmentation for other organs.
Jinzhu et al [27]	3D U-Net with ResNet model	Precision, sensitivity, and maximum dice coefficient were achieved.	The potential for extending MSDS-UNet to utilize multiple advanced encoder backbones like UNet++ or HRNet was not explored.
Shimpy Goyal and Rajiv Singh [28]	ANN SVM KNN	It provides low computational efforts with better accuracy.	The proposed work requires augmentation concerning severity analysis and adaptive model construction, particularly for incorporating supplementary datasets encompassing multiple classes.
Min Hong, et al. [29]	CNN	It attained average test inference time.	Need to explore learning techniques for a broader range of strategies and disease types for enhancing learning efficiency.

Nevertheless, clinicians must differentiate between various infected areas to assess the condition accurately. The LGAN model was deployed in [26] and provides improved robustness and better Dice score; however, there is a need to expand the proposed framework to include segmentation for other organs. 3D U-Net equipped with a ResNet model was presented in [27], offering precision, sensitivity, and maximum dice coefficient; however, the potential for extending MSDS-UNet to utilize multiple advanced encoder backbones like UNet++ or HRNet was not explored. Thus, the limitations of using CT images effectively to improve lung segmentation in the current research must be considered.

## 3 An overview of M-Segnet-based lung disease segmentation and severity classification using SDPA–Squeezenet model

### 3.1 Proposed framework model

**Fig 1:**
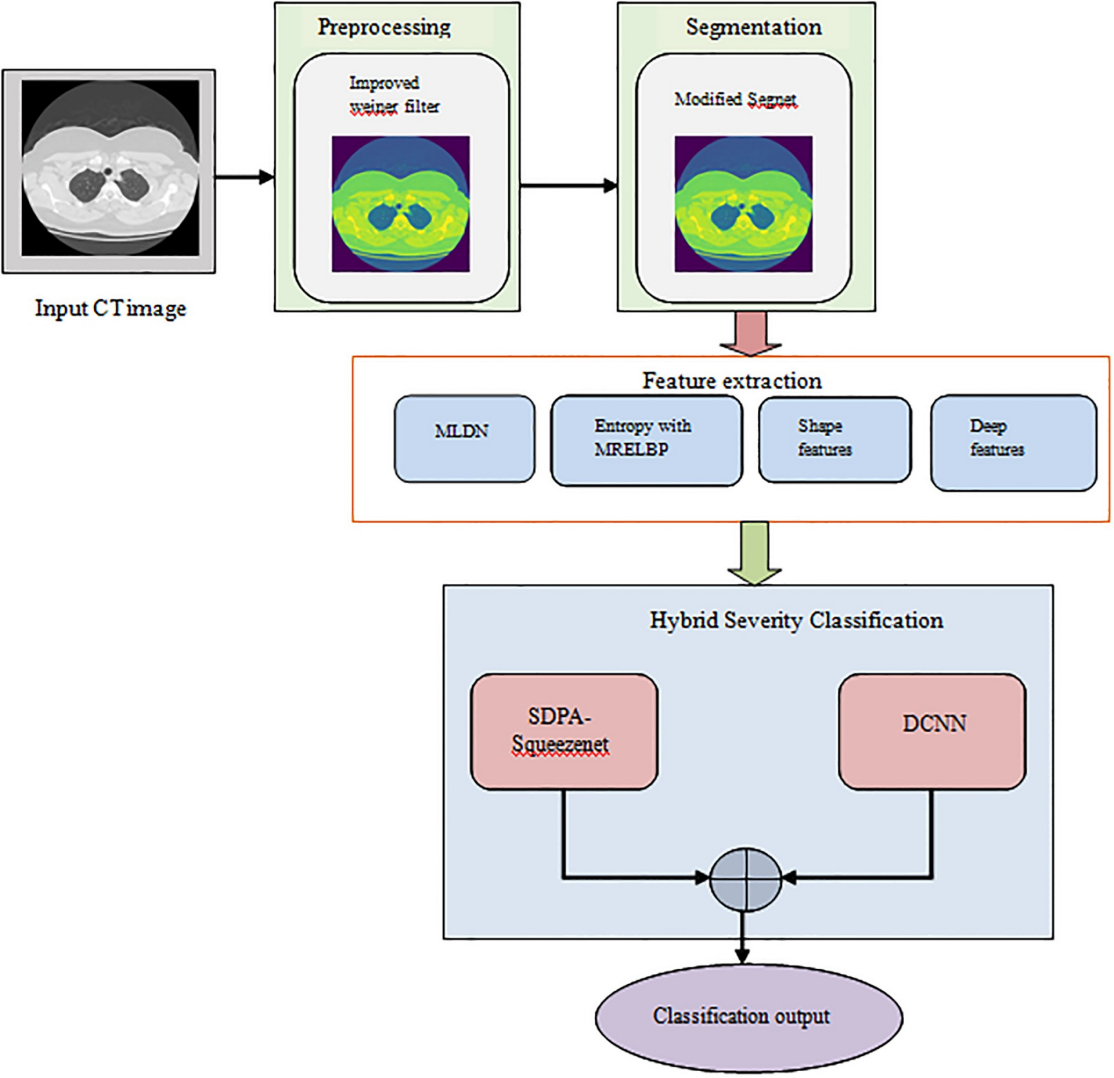
Framework of MSLDSSC model.

Lung diseases, encompassing a broad spectrum from infections to chronic conditions like COPD and lung cancer, pose significant challenges to global healthcare systems. Accurate and timely diagnosis is paramount for effective treatment planning, monitoring disease progression, and improving patient outcomes. In recent years, integrating advanced imaging techniques with machine learning methodologies has shown promising results in automating the diagnosis and management of lung diseases. This paper proposes a novel Modified Segnet-based Lung Disease Segmentation and Severity Classification (MSLDSSC) framework, as illustrated in Fig 1. The proposed MSLDSSC model has four phases: "preprocessing, segmentation, feature extraction, and classification." Initially, the input image is preprocessed using an improved Wiener filter technique. This technique estimates the power spectral density of the noisy and original image, and PSNR-assisted SNR is computed to estimate the image quality.

Further, the preprocessed image is segmented to identify and separate the RoI from the background objects in the preprocessed lung image. We adopt a Modified Segnet mechanism that uses the proposed hard tanh-Softplus activation function to segment effectively. Subsequently, the features such as MLDN, entropy with MRELBP, shape features, and deep features are retrieved. The retrieved feature set is passed into the hybrid severity classification model after the feature extraction phase. The hybrid severity classification model encompasses SDPA-Squeezenet and DCNN classifiers. This hybrid classification model trains the retrieved feature set and effectively classifies the lung severity level.

### 3.2 Preprocessing the original image using an improved Wiener filter

Images captured by cameras or generated by sensors often contain noise, which can distort the true information in the image. Preprocessing techniques like smoothing filters can help reduce noise, improving the quality of the image. Consider *Img* the input image subjected to the preprocessing technique to remove the noise *Img*. We adopt the Improved Wiener filter technique for preprocessing the CT images when the noise characteristics are known or can be estimated accurately. The elucidation of the Improved Wiener filter technique is as follows:

Improved Wiener filter technique: Wiener filtering [30] for additive noise removal involves employing low-pass filters that adapt to individual pixels based on information gathered from their local neighborhoods. It presumes an understanding or estimation of both the inherent traits of the initial image and the noise distorting it. This method computes the local mean and variance during the filtering process. It conducts de-convolution through inverse filtering and eliminates noise via a compression operation. The prescribed procedure typically involves the following standard steps:

Step 1: Compute the power spectra of the noisy and original image by employing the Fourier transform of the autocorrelation function.
Step 2: Place a mask onto a pixel within a noisy image.
Step 3: Evaluate the variance and local mean.
Step 4: Estimate new value for the pixel by adopting variance, noise power, and mean.
Step 5: Reiterate steps 2 to 4 for every noisy image pixel.

While Wiener filtering is a powerful technique for image restoration and noise reduction, its effectiveness can be limited by the assumptions it makes, and it can be computationally intensive, especially for large images. Wiener filtering assumes prior knowledge or assumptions about both the image and the noise. Accurate knowledge of the noise characteristics may not always be available or feasible. Wiener filtering can introduce artifacts or distortions, such as ringing or overshooting near sharp edges or transitions. These artifacts affect image quality and visual perception. An improved wiener filtering technique is used to tackle this issue. The improved Wiener filter achieves significantly better denoising and restoration compared to the original MSE-optimized counterpart. It preserves signal features while reducing noise effects. The improved Wiener filter still optimizes the MSE but considers alternative metrics like Structural Similarity (SSIM). SSIM provides a more accurate assessment of image similarity and quality. The steps to be followed for the improved wiener filter are as follows:

Step 1: Compute the power spectral density of the noisy and original image.
Step 2: Further compute PSNR [31] along with SNR as in Eq (1), which is deployed to estimate the quality of the image.
$$I_{\left(PSNR_{New}*SNR_{New}\right)}=\left\{10{\text{log}}_{10}\left[\frac{255^{2}\times P\times Q+\left[E\prime \left(i,j\right)+E_{0}\left(i,j\right)\right]}{\sqrt{\sum_{i=1}^{Q}\sum_{j=1}^{Q}{\left|E\prime \left(i,j\right)+E_{0}\left(i,j\right)\right|}^{2}}*\sqrt{\sum_{i=1}^{Q}\sum_{j=1}^{Q}{\left(E_{0}\left(i,j\right)\right)}^{2}}}\right]*10\text{log}\left[\frac{{\left|H_{Mean}\right|}^{2}}{2\times Var}\right]\right\}$$ (1)
  where, $Var=\frac{1}{Q}\sum_{n=1}^{Q}{\left(\left|x_{n}\right|-H_{Mean}\right)}^{2}$, *H*_*Mean*_ indicates harmonic mean, *P* and *Q* matrix dimension of *Img*, *E*_0_(*i*, *j*) indicates the pixels’ gray value in *j*^*th*^ the column and *i*^*th*^ row of the original image and *E*′(*i*, *j*) indicates the pixels’ gray value in *j*^*th*^ the column and *i*^*th*^ row of the restored image.
Step 3: Place a mask onto a pixel within a noisy image.
Step 4: Arrange each pixel’s intensities that fall under the mask.
Step 5: Estimate the median value and allot it to the masks’ central pixel.
Step 6: Compute variance and mean value as in Eqs (2) and (3), respectively.
$$Var\left(\sigma^{2}\right)=\frac{1}{uv}{\left(Img\left(u,v\right)-\mu \right)}^{2},$$ (2)
  where *u* and *v* indicates the row and column of *Img*.
$$Mean\left(\mu \right)=\frac{1}{uv}\sum_{uv}Img\left(u,v\right)$$ (3)
Step 7: Estimate new value for the pixel *S*(*u*, *v*) as in Eq (4).
$$S\left(u,v\right)=\left\{\text{tanh}\left[Med+\frac{\sigma^{2}-V^{2}}{\sigma^{2}}\left(Img\left(u,v\right)-Med\right)\right]+1\right\}$$ (4)
  where, *Med* indicates median value and *V*^2^ indicates noise variance.
Step 8: Reiterate steps 2 to 7 for every noisy image pixel.

Thus, the preprocessed image can be denoted by *Img*^*pre*^.

### 3.3 Segmentation

The segmentation process in lung disease classification can be partitioning an image into multiple segments based on certain characteristics such as color, intensity, or texture. Segmentation is a fundamental step in many image processing tasks, and there are various techniques to simplify the image representation into something more essential and easier to analyze. This work adopts a modified Segnet approach for segmenting the preprocessed image *Img*^*pre*^. The procedure of modified Segnet is as follows:

Modified Segnet: Segnet [32] is a specialized deep learning architecture crafted specifically for semantic segmentation tasks, utilizing an encoder-decoder structure comprising CNNs and pooling layers to classify image pixels. In SegNet’s encoder component, multiple convolutional layers are followed by pooling layers that extract features with high levels from the input image while decreasing spatial dimensions through downsampling operations, gradually building hierarchical representations. Conversely, the decoder portion mirrors the encoder’s layout but performs upsampling instead of downsampling, refining feature representations and generating pixel-wise segmentation masks. Skip connections between corresponding layers aid in preserving spatial information lost during encoder downsampling, enhancing segmentation accuracy by amalgamating features from various abstraction levels. Finally, a softmax layer is applied to each pixel in the output feature map, assigning a probability distribution across different classes and yielding the final segmented image. However, SegNet uses pooling indices from the encoder during decoding for non-linear upsampling. While this approach is memory-efficient, it may not handle complex spatial relationships well. The decoder’s requirement to store feature maps at multiple resolutions during upsampling can lead to high memory consumption, prompting the proposal of a modified SegNet framework to mitigate this challenge. The modified SegNet model is memory-efficient and computationally faster during inference. It optimizes resource usage while maintaining segmentation accuracy. The modified SegNet framework consists of a hierarchical correspondence of encode-decoder layers. This design facilitates feature extraction and context preservation during segmentation.

**Fig 2:**
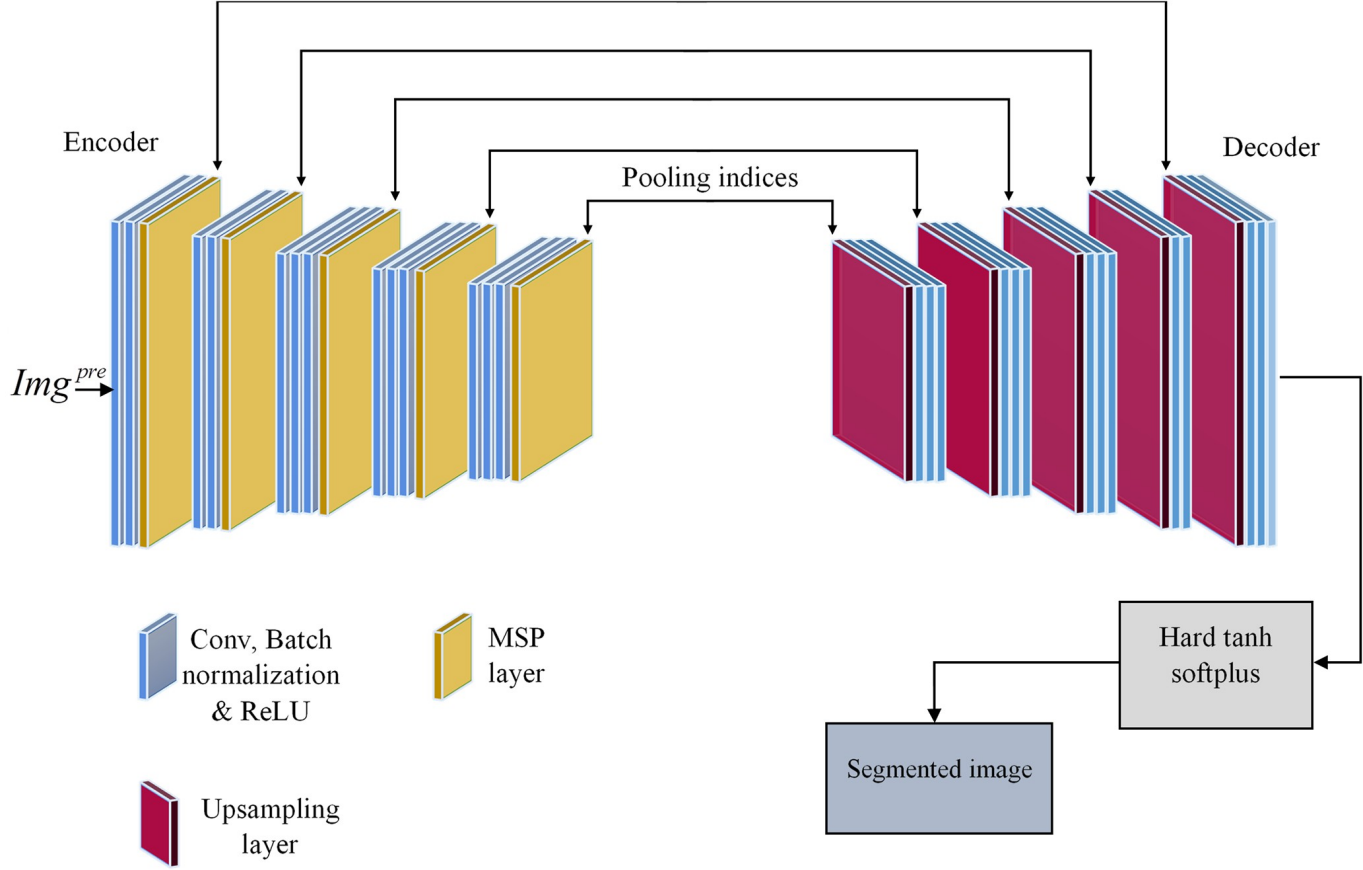
Modified Segnet framework.

As illustrated in Fig 2, the proposed modified Segnet framework [33] is structured into an encoder and decoder, each consisting of 13 convolutional layers. Initially, the encoder layers utilize a small receptive field of two convolution layers with a size of 64×64. They incorporate ReLU activation functions along with BN operations. Following the convolutional layers, the mixed stochastic pooling operations are performed to maintain the spatial dimensions of the feature maps. Likewise, the convolution layer with size 128x128 is applied for two layers, the convolution layer with size 256x256 is applied for three layers, the convolution layer with size 512x512 is applied for three layers, and the convolution layer with size 512x512 is applied for three layers. After these convolutional layers, mixed stochastic pooling operations are performed. This work utilizes five mixed stochastic pooling operations defined as in Eq (5). Here, *γ* = 1.


$$MSpool_{j}=\left[\left[\gamma \underset{i\in R_{j}}{Max}a_{i}+\left(1-\gamma \right)\frac{1}{\left|R_{j}\right|}\sum_{i\in R_{j}}a_{i}\right]+a_{l}\right]$$ (5)

In stochastic pooling, the probability *p*_*i*_ is evaluated at first as in Eq (6). Such probabilities generate a multinormal distribution, which is adopted to choose a location *l* as well as the relevant pooled activation *a*_1_ according to *p* multinormal distribute chooses a location *l* in the region as in Eq (7). Here, *a*_*i*_ indicates feature value at local *i* in the pooling region *R*_*j*_.


$$p_{i}=\frac{a_{i}}{\sum_{k\in R_{j}}a_{k}}$$ (6)

$$MSpool_{j}=a_{l}\;,\;\;\text{where}\;l∼p\left(p_{1},\ldots ,p\left|R_{j}\right|\right).$$ (7)

On the other hand, the decoder mirrors the encoder that performs similar operations except for the mixed stochastic pooling (MSP) operation. Instead of a mixed stochastic pooling operation, upsampling is performed in the decoder part. Consequently, a new hard tanh softplus activation function is applied instead of the softmax activation function. The hard tanh activation function, a modified version of the conventional hyperbolic tangent (tanh) function employed in neural networks, adjusts its behavior by limiting output beyond specific thresholds, typically -1 and 1. Unlike the tanh function, which outputs values within the range of -1 to 1, the hard tanh function restricts values beyond these limits. This adaptation enhances the function’s resilience to extreme inputs, stabilizing training processes within deep neural networks. Thus, the hard tanh activation function [34] is defined as in Eq (8).


$$f\left(x\right)=\left(\begin{array}{lll} -1 & if & x<-1\\ x & if & -1=x\leq 1\\ 1 & if & x>1 \end{array}\right)$$ (8)

However, the conventional hard tanh activation function suffers from gradient saturation issues. A new hard tanh soft plus activation function is used to overcome this issue, as defined in Eq (9).


$$f\left(x\right)=\left(\begin{array}{l} -1\;\;\;\;if\;\;\;x<-1\\ \left[\frac{x}{\left|x\right|+1}\right]\;\;\;\;\;\;if\;\;\;-1=x\leq 1\\ 1\;\;\;\;\;\;if\;\;\;\;x>1 \end{array}\right)+\text{log}\left(1+{\text{exp}}^{x}\right)$$ (9)

Thereby, the segmented image is denoted by *Img*^*seg*^.

### 3.4 Feature extraction: MLDN, MRELBP, shape features, and deep features

After applying the segmentation process, the segmented image *Img*^*seg*^ is subjected to the feature extraction procedure. The features, including MLDN, MRELBP, shape, and Deep features are retrieved from the segment *Img*^*seg*^ to retrieve relevant information. This process is crucial for reducing the dimensionality of the data and extracting meaningful patterns; it is used for further analysis. There are various methods for feature extraction. This work extracts features like MLDN, MRELBP, shape features, and deep features for retrieving the frequency of different patterns, capturing the distribution of texture patterns’ shape features, and capturing more abstract and high-level visual concepts.

#### 3.4.1 MLDN

The MLDN employs two distinct asymmetric masks: the Kirsch and derivative-Gaussian masks. These masks are designed to operate within the gradient space, thereby revealing the facial structure. Additionally, Gaussian smoothing is utilized to improve the stability of the code in the presence of noise, specifically by applying the derivative Gaussian mask.

The Kirsch mask is rotated 45 degrees to capture edge responses in eight directions. This mask is denoted as LDNK, which is utilized to generate the LDN [35] code. Additionally, the derivative of a skewed Gaussian is used to develop an asymmetric compass mask. This mask is applied to compute edge responses on the smoothed face. Notably, it exhibits robustness against illumination and noise variations while yielding prominent edge detection outcomes. The Gaussian mask mentioned is defined as in Eq (10). Here, *σ* it denotes Gaussian bell width and *p*, *q* denotes the position of locations.


$$g_{\sigma }\left(p,q\right)=\frac{1}{2\pi \sigma^{2}}\text{exp}\left(-\frac{p^{2}+q^{2}}{2\sigma^{2}}\right)$$ (10)

Gaussian masks tend to produce smoother results by blurring edges. In LDN, this blurring effect can lead to edge detail loss, reducing-edge detection’s effectiveness. LDN requires prior knowledge or assumptions about the local neighborhood’s directional information. Accurate estimation of directional patterns may be challenging in noisy or complex environments. LDN’s performance can degrade in the presence of noise or variations in illumination, pose, or expression. Robustness to these factors is essential for reliable feature extraction. Hence, a new formulation of the Gaussian mask is improved, as in Eq (11).

$$Impg_{\sigma }\left(p,q\right)=\frac{1}{2\pi \sigma^{2}}\text{exp}\left(-\frac{p^{2}+q^{2}}{2\sigma^{2}}\right)*\frac{1}{1+e^{-{\left(p_{i}+q_{i}\right)}^{2}/2\left(p_{i}+q_{i}\right)}}$$ (11)

Where, *σ* denotes standard deviation and the standard form of defining the mask can be defined as in Eq (12). Then the modified form of defining mask is defined as in Eq (13). Here, *σ* signifies the width of the Gaussian bell, *g*_*σ*_ represents the Gaussian function, * indicates convolution operator and *k* denotes the offset of the Gaussian from its center.


$$M_{\sigma }\left(p,q\right)=g_{\sigma }^{\prime }\left(p+k,q\right)*g_{\sigma }\left(p,q\right)$$ (12)

$$M_{\sigma }{\left(p,q\right)}_{Imp}=MED\left(g_{\sigma }^{\prime }\left(p+k,q\right)\right)*Impg_{\sigma }\left(p,q\right)$$ (13)

Thereby, the extracted MLDN-based features can be denoted as *Mldn*^*f*^. MLDN consistently performs well under variations in illumination, noise, expression, and time lapse. Its robustness ensures reliable recognition across different conditions. MLDN divides the face into several regions and extracts the distribution of features from each region. Concatenating these region-based features into a single descriptor enhances the overall representation.

#### 3.4.2 Entropy with MRELBP

The entropy with MRELBP-based features is extracted from *Img*^*seg*^. Initially, the mean is computed for the image *Img*^*seg*^. Then, apply LBP, a texture descriptor used in image processing, to classify textures by comparing pixels with their neighbors, generating binary patterns that describe the texture. The median robust extension of LBP [36] enhances its robustness to noise and outliers by incorporating median filtering into its computation. MRELBP is inherently robust to image noise, as it compares regional image medians rather than raw pixel intensities. When combined with entropy, it further enhances noise resilience by capturing the distribution of pixel values in a more informative way.

The LBP operator is defined as in Eq (14).


$$Lbp_{x,y}\left(z_{c}\right)=\sum_{n=0}^{y-1}s\left(z_{x,y,n}-z_{c}\right)2^{n}\;$$ (14)

Here,$s\left(i\right)=\left\{\begin{array}{l} 0,\;\;\;\;\;z<0\\ 1,\;\;\;\;\;\;z\geq 0 \end{array}\right.$. Then, apply a histogram to summarize the distribution of the local binary patterns within an image. Histograms of LBP patterns encode the information relevant to an image’s texture. By analyzing the distribution of patterns, we can capture the texture characteristics such as smoothness, roughness, or regularity. Also, the dimensionality of the feature space is reduced.

Further, the output from the histogram is subjected to perform entropy. In image processing, entropy is a metric for the level of uncertainty or randomness present in a variable. Specifically, it is frequently utilized as a texture descriptor to gauge the extent of information or disorder contained within an image. The entropy [37] is defined as in Eq (15). Here, *Sp*_*i*_ it indicates sampling pixels’ probability.


$$H\left(Sp_{i}\right)={\text{log}}_{2}\frac{1}{P\left(Sp_{i}\right)}$$ (15)

Thereby, the entropy with the MRELBP-based feature extracted can be denoted by *Elbp*^*f*^.

#### 3.4.3 Shape features

The shape feature [38] is extracted *Img*^*seg*^ to retrieve valuable information about objects’ spatial characteristics and geometric properties within the image. This work considers specific characteristics like area, perimeter, and convex hull.

**Area**: It refers to the total number of pixels within the segmented image, serving as a metric of its size.

**Perimeter**: It indicates the length of the boundary surrounding the segmented object, offering insight into the outer boundary length of the object.

**Convex hull**: The convex hull of the smallest convex set containing all points within a set in Euclidean space. It can be visualized as the shape formed by stretching a rubber band around the outermost points of the set.

Thus, the extracted shape-based features can be denoted by *S*^*f*^.

#### 3.4.4 Deep features

Deep features refer to high-level representations of data learned by deep learning models, particularly DNNs. These features capture complex patterns and structures in the input data, allowing the model to make more accurate predictions or classifications. Deep features are powerful because they encode rich semantic information about the input data, enabling the model to effectively understand and generalize complex patterns. In this work, two deep learning models, like ResNet and VGG16, learn the data.

ResNet: Residual Network [39] is a type of DCNN designed to address the challenge of vanishing gradients in training deep neural networks. This problem arises when adding more layers to traditional networks, causing gradients to become extremely small and hindering effective training. ResNet resolves this issue by introducing skip or shortcut connections, which allow gradients to flow more easily by bypassing one or more layers. This enables the training of very deep networks more effectively. The fundamental component of ResNet is the residual block, comprising multiple convolutional layers followed by BN and ReLU activation functions.

Moreover, the output of the block is achieved by summing the input with the output generated from the convolutional layers, allowing the network to learn residuals or the difference between input and desired output. This approach facilitates the training of deeper network1+s by enabling them to focus on learning residual information rather than directly mapping input to output. The extracted ResNet features can be denoted by *Res*^*f*^.

VGG16: VGG16 [40], developed by the Visual Geometry Group (VGG) at the University of Oxford, is DCNN architecture renowned for its 16-layer depth. It comprises 13 convolutional (Conv) layers and 3 fully connected layers, distinguished by its uniform and straightforward design. Featuring 3x3 Conv filters throughout, VGG16 excels in utilizing deep Conv layers for feature extraction, complemented by fully connected layers for classification. Its architecture consists of blocks of Conv layers followed by max-pooling layers to decrease spatial dimensions and enhance the receptive field. The fully connected layers ultimately perform the conclusive classification based on the extracted features. The extracted VGG16 features can be denoted by *Vgg*^*f*^.

Thereby, the extracted deep features can be represented, and the overall feature set extracted from *Img*^*seg*^ can be denoted by *f*_*set*_ = [*Mldn*^*f*^, *Elbp*^*f*^, *s*^*f*^, *D*^*f*^].

### 3.5 Hybrid severity classification model

The hybrid model achieves impressive classification accuracy, which is crucial for reliable disease detection. By leveraging the strengths of both SDPA-SqueezeNet and DCNN, it can accurately differentiate between different lung conditions. SqueezeNet is known for its lightweight architecture, making it computationally efficient. By integrating it with DCNN, which provides deeper feature extraction, the hybrid model balances efficiency and performance. SDPA-SqueezeNet and DCNN together handle variations in lung images due to factors like lighting, positioning, and patient-specific characteristics. The hybrid model can generalize well to unseen data. It learns from a wide range of lung images, allowing it to adapt to new cases and improve overall diagnostic accuracy. The extracted feature set *f*_*set*_ is passed into these two models individually and trains the feature set. Further, these two models provide classified output, and their output’s average determines the classified output.

#### 3.5.1 SDPA- Squeezenet

**Fig 3:**
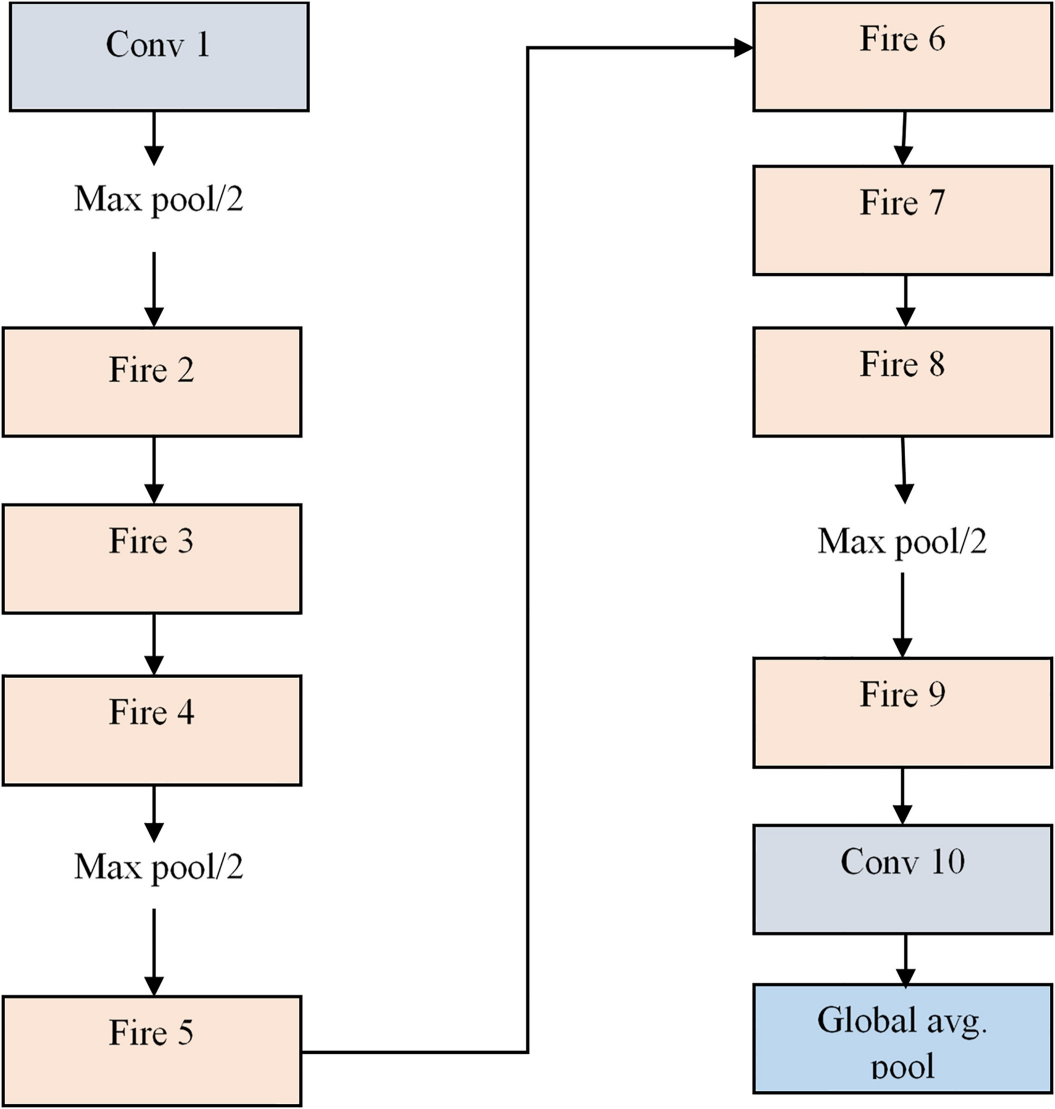
Standard form of SqueezeNet model.

SqueezeNe [41] is an innovative CNN architecture that aims to attain high accuracy while minimizing computational demands. Its pioneering feature lies in the utilization of "fire" modules, comprising squeeze layers (1x1 convolutions) to diminish input channels and expand layers (1x1 and 3x3 convolutions) to augment output channels. This approach markedly reduces parameters compared to conventional CNNs, enhancing efficiency in memory and computation. Moreover, SqueezeNet employs strategies such as global average pooling and ReLU activation functions to further diminish the model size and intricacy. As depicted in Fig 3, the structure of Squeezenet involves a convolutional layer that takes the extracted feature set as input and performs average max pooling; following this, fire modules like Fire 2, Fire 3, and Fire 4 are applied, and again, it performs average max pooling. Subsequently, the output from max pooling is passed into the fire modules like Fire 5, Fire 6, Fire 7, and Fire 8, and then it performs average max pooling. Further, the output from max pooling undergoes Fire 9 and then the convolution layer. Finally, it conducts global average pooling, softmax activation function, and categorical cross-entropy loss function, which is applied at the output layer.

**Fig 4:**
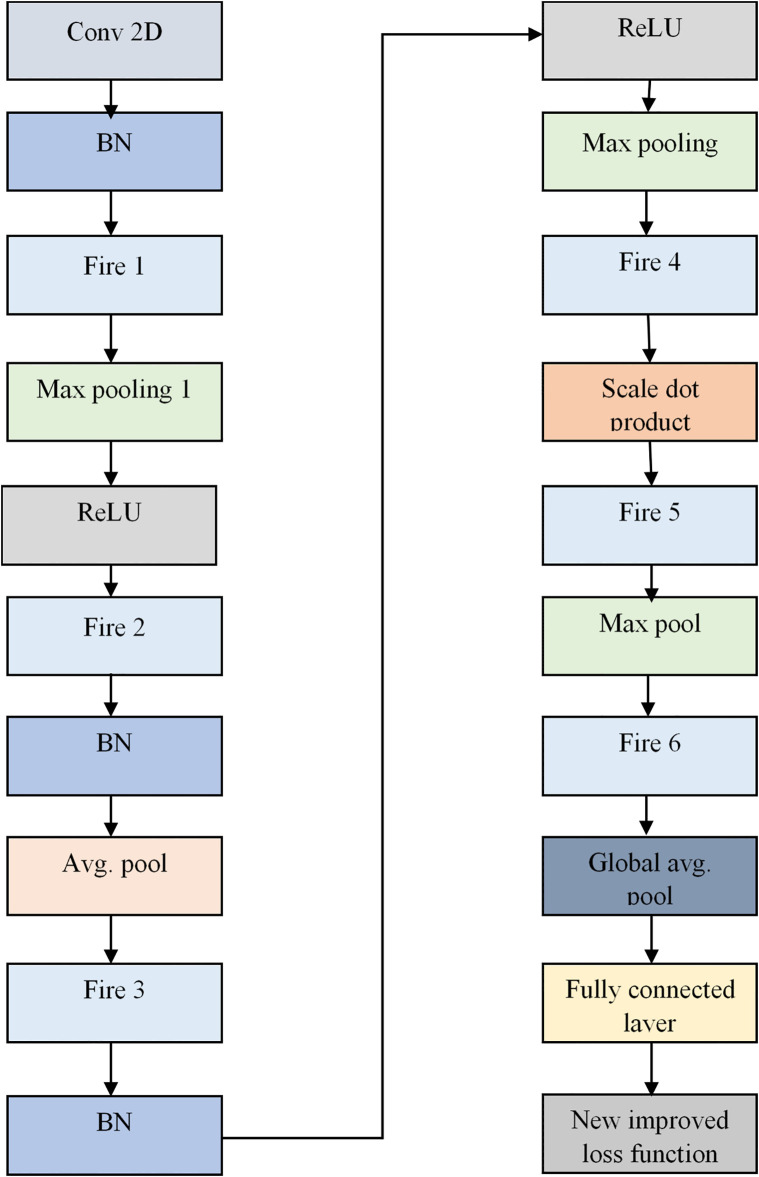
Structural representation of SDPA-SqueezeNet model.

This standard form of SqueezeNet involves reducing the number of channels before applying convolution, which may lead to information loss. This bottleneck could limit the model’s ability to capture intricate details in the data. A new Scale Dot Product Attention-based Squeezenet (SDPA-SqueezeNet) model is proposed to overcome this issue. The structure of the SDPA-SqueezeNet model is illustrated in Fig 4. As shown in Fig 4, The extracted feature set is passed into the Conv-2D layer that convolves the input data with learnable filters to produce feature maps and perform Batch normalization that normalizes each layer’s inputs. It addresses the issue of internal covariate shift, which refers to the fluctuation in the distribution of the inputs to a layer as the parameters of the prior layers change during training. Following this, the Fire 1 module is performed to capture channel-wise and spatial correlations within the input data while minimizing computational complexity and model size. Then, the max pooling operation reduces their spatial dimensions while retaining important information and applies the ReLU activation function and Batch Normalization, enhancing feature extraction and dimensionality reduction. The Fire modules, consisting of squeeze and expand layers, efficiently capture channel-wise and spatial correlations within the input data, which is crucial for effective feature extraction. The architecture incorporates average pooling to further down-sample feature maps and a scaled dot product attention layer to enhance the model’s ability to focus on important features. Following additional Max Pooling and Fire modules, the network employs a global pooling layer to aggregate features across the spatial dimensions. Finally, fully connected layers process the extracted features for classification, followed by an Improved loss function to compute the discrepancy between predicted and actual labels, guiding the model’s optimization during training.

Scale dot product attention: It can capture long-range dependencies in sequential input data. The scaled dot product attention [42] is defined as in Eq (16).

$$Att\left(Q,k,V\right)=Soft\text{max}\left(\frac{Qk^{T}}{\sqrt{dk}}\right)V$$ (16)

Where *V* denotes the actual value behind the key, *Q*, *k* indicates two matrices *Q*(*query*) *k*(*query*), respectively.

Improved loss function: It measures the discrepancy between the true class labels and the predicted probabilities assigned by the model. This model utilizes a hybrid activation function for effectiveness. The hybrid loss function involves two activation functions, log sig and hyperbolic activation function. Then, the hybrid improved loss function [43] can be expressed as in Eqs (17) and (18).

$$f\left(x\right)=\frac{1}{1+e^{-x}}+\frac{e^{x}-e^{-x}}{e^{x}+e^{-x}}$$ (17)

$$\;\;\;\;\;\;\;=\left[\frac{e^{x}+e^{-x}+\left(1+e^{x}\right)\left(e^{x}-e^{-x}\right)}{\left(1+e^{x}\right)\left(e^{x}-e^{-x}\right)}\right]$$ (18)

where, $\frac{1}{1+e^{-x}}$ indicates log sig activation function and $\frac{e^{x}-e^{-x}}{e^{x}+e^{-x}}$ indicates hyperbolic activation function. Again, this loss function undergoes Tversky loss function derivation [44] as in Eqs (19) and (20). Here, *β* = 1/2.


$$f^{\prime \prime }\left(x\right)=Tversky\;loss\left(f\left(x\right)\right)$$ (19)

$$\;\;\;\;\;\;\;\;\;\;\;\;\;\;=\frac{p\hat{p}}{p\hat{p}+\chi \left(1-p\right)\hat{p}+\left(1-\chi \right)p\left(1-\hat{p}\right)}\left(f\left(x\right)\right)$$ (20)

Thereby, the result obtained from SDPA-SqueezeNet is denoted as $S_{Sdpa}^{Out}$.

#### 3.5.2 DCNN

DCNN [45] is a neural network specifically designed to process visual data, such as images. It comprises multiple layers, including Conv, pooling, and fully connected layers.

Convolutional layers are responsible for retrieving features from the input data by performing filters across the image. These filters detect patterns at different spatial locations, such as edges or textures. Moreover, the Pooling layers reduce the spatial dimensions of the feature maps produced by the Conv layers. This helps decrease the network’s computational complexity while retaining important features. Consequently, the fully connected layers perform the final classification based on the features extracted by the Conv and pooling layers. They combine and interpret the features to produce the desired output *Dcnn*^*out*^.

**Final classification**: Thus, the output from SDPA-SqueezeNet and DCNN model like $S_{Sdpa}^{Out}$, and *Dcnn*^*out*^, respectively, are determined to take average, and the average result classifies the lung disease as ’0-Negative, 1-Air space consolidation, 2-Crazy paving and 3-GGO’.

## 4 Results and discussion

### 4.1 Simulation setup

The proposed Lung Disease Segmentation and Severity Classification using CT images were implemented using PYTHON, and its version was "Python 3.7". The simulation was conducted on a processor with an "Intel(IR) Core(TM) i5-4210U CPU @ 1.70GHz 1.70 GHz,", the system was equipped with "8.00 GB" of installed RAM, and the system type was 64- bit operating system, x64- based processor. Also, the edition of window was Windows 11 Pro and its version was 22 H 2.

### 4.2 Dataset description

**Fig 5:**
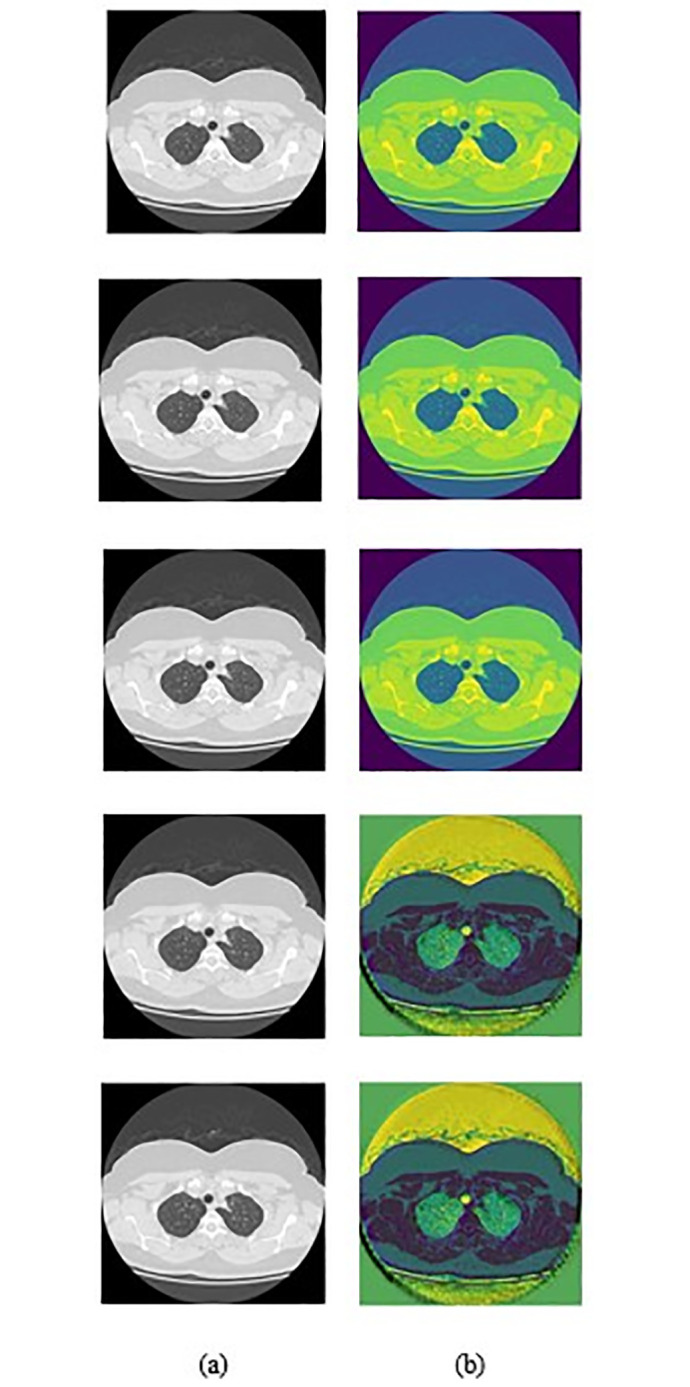
Image Results for Lung Disease Segmentation and Severity Classification using CT Images a) Input Images and b) Improved Weiner Filtering.

The examination of lung disease segmentation and severity classification using CT images was conducted with the HRCTCov19 dataset [46]. This dataset, encompassing slice- and patient-level labeling, holds significant promise for COVID-19 research, especially in diagnosis and differentiation through AI algorithms, machine learning, and deep learning methodologies. There are total 3345 images in this dataset. Here, 3D images are used for training the dataset. There are four labels namely, 1 denotes Air Space Consolidation, 2 denotes Crazy Paving, 3 denotes GGO, 0 denotes negative. Table 2 describes the training and testing images for HRCTCov19 dataset. Fig 5 illustrates the original and Improved Weiner Filtering-based preprocessed images.

**Table 2:** Training and testing images for HRCTCov19 dataset.

Percentage	Training images	Testing images
60	2007	1338
70	2341	1004
80	2676	669
90	3010	335

### 4.3 Segmentation analysis

**Fig 6:**
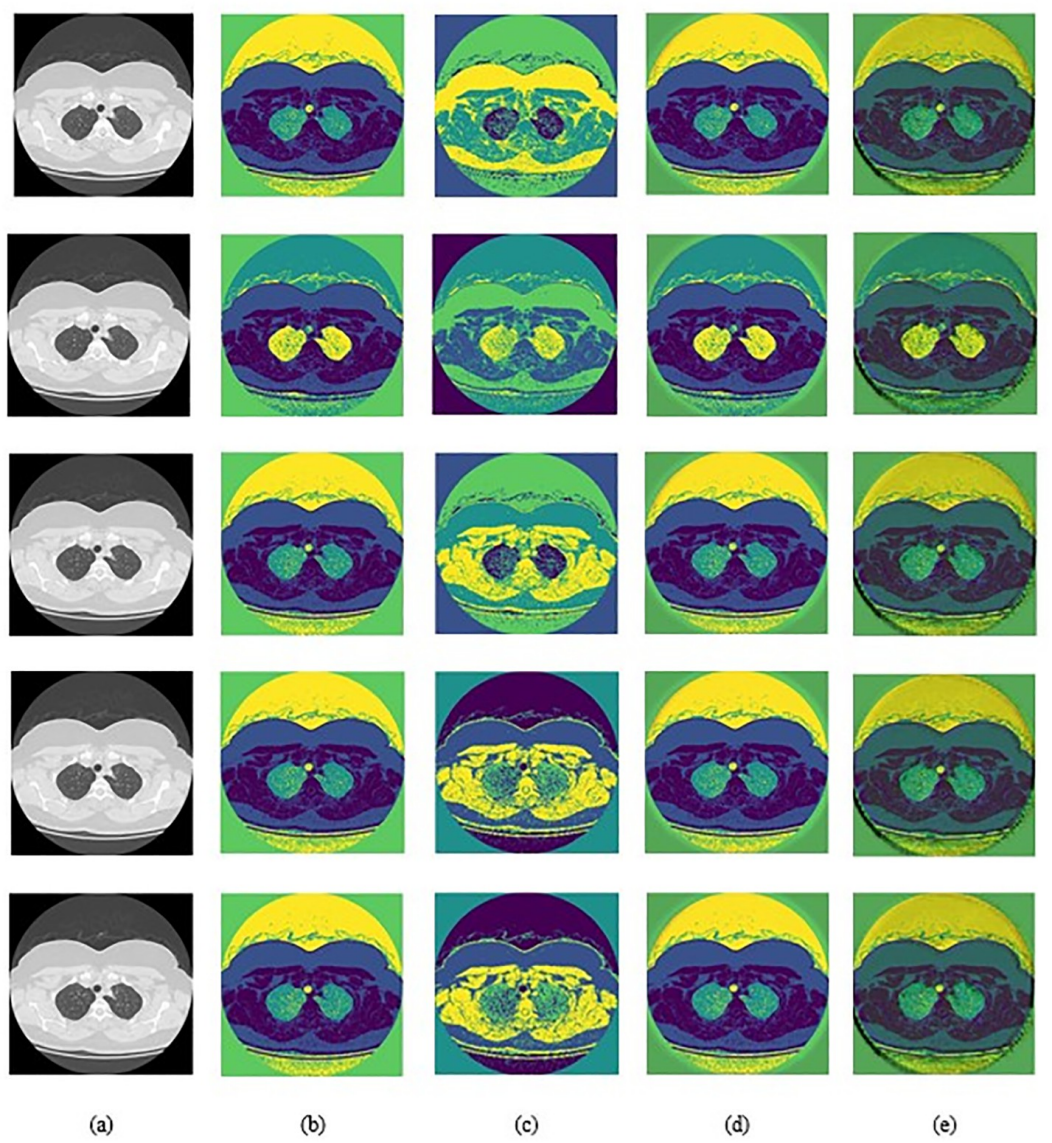
Image Results for Lung Disease Segmentation using CT images a) Input Image b) FCM c) nnU-Net d) K-Means e) Conventional SegNet and f) Modified SegNet.

The comparative analysis of segmentation techniques is conducted, particularly focusing on Modified SegNet alongside traditional segmentation methods for lung disease segmentation using CT Images. Fig 6 showcases the input images and their corresponding segmented results obtained through FCM, K-means, nnU-Net, Conventional SegNet, and Modified SegNet. Impressively, Modified SegNet exhibits superior segmentation outcomes when compared to traditional methods. Additionally, Table 3 provides a detailed assessment of segmentation accuracy, Dice coefficient, and Jaccard coefficient for both Modified SegNet and conventional strategies. This comprehensive evaluation offers valuable insights into the effectiveness of Modified SegNet in accurately delineating lung disease regions, highlighting its potential for improved diagnostic applications. "The Dice coefficient, also known as the Dice similarity coefficient or Dice index, is a statistical measure used to quantify the similarity between two data sets." The following expression defines it:

$$DSC(A,B)=\frac{2(A\cap B)}{\left(A+B\right)}$$ (21)

**Table 3:** Segmentation analysis on modified SegNet over conventional approaches.

Metrics	FCM	Modified SegNet	K-MEANS	nnU-Net	Conventional SegNet
**Dice**	0.687	0.876	0.692	0.684	0.745
**Jaccard**	0.675	0.953	0.687	0.695	0.839
**Segmentation Accuracy**	0.674	0.962	0.677	0.675	0.841

In the realm of image segmentation evaluation, "A" symbolizes the "segmented image," whereas "B" denotes the "predicted image." Similarly, "The Jaccard coefficient, also known as the Jaccard similarity coefficient or Jaccard index, is a statistical measure used to evaluate the similarity and intersection between two sets of data." Besides, it can be stated as:

$$J(A,B)=\frac{\left|A\cap B\right|}{\left|A\cup B\right|}$$ (22)

Table 3 demonstrates that the Modified SegNet achieved a segmentation accuracy of 0.962, indicating superior performance compared to the FCM (0.674), nnU-Net (0.675), K-Means (0.677), and Conventional SegNet (0.841) models. Notably, these traditional methods lagged behind the Modified SegNet regarding segmentation accuracy. Similarly, the Jaccard and Dice coefficients attained by the Modified SegNet markedly exceed those attained by the established segmentation approaches. Furthermore, the substantial advancement in Segmentation accomplished by the Modified SegNet suggests a more precise delineation of lung disease regions. This heightened segmentation accuracy enhances the identification of affected areas and sets the stage for a more precise classification of lung disease severity. Table 4 describes the statistical analysis for the segmentation approaches under diverse measures. Statistical analysis entails evaluating numerous iterations of diverse models, each iterations executed multiple times. Throughout this procedure, analysts compute statistical metrics such as mean, median, maximum, minimum, and standard deviation, applying them once to the results of these runs. The modified SegNet achieves a rate of 0.9678 in maximum measures which is superior when compared to the nnU-Net, FCM, K-Means and conventional SegNet. Similarly, in other statistical measures, the modified SegNet approach acquired greater ratings like 0.9595, 0.9623, 0.9610, and 0.0032. Thus the modified SegNet approach shows its better efficacy in statistical measures for the segmenting the preprocessed images for lung disease classification.

**Table 4:** Statistical analysis for segmentation approaches.

Segmentation Approaches	Minimum	Maximum	Mean	Median	Standard deviation
nnU-net	0.819418	0.830915	0.824873	0.824579	0.005318
FCM	0.653271	0.694002	0.674498	0.67536	0.014612
K- Means	0.667506	0.694778	0.676825	0.672508	0.010609
Conventional SegNet	0.822822	0.85441	0.840902	0.843187	0.011614
Modified SegNet	0.959589	0.967863	0.962398	0.96107	0.003238

### 4.4 Performance analysis

A thorough comparative analysis was undertaken to assess the performance of the SDPA-SqueezeNet+DCNN scheme within the realm of Lung Disease Segmentation and Severity Classification utilizing CT images, compared to conventional approaches. This thorough evaluation encompassed key metrics such as "Sensitivity, False Negative Rate (FNR), Negative Predictive Value (NPV), Specificity, F-measure, Precision, False Positive Rate (FPR), Matthews Correlation Coefficient (MCC), and Accuracy." Additionally, the assessment included analyses of Statistical Analysis, Ablation Study, and Confusion Matrix. Moreover, the performance of the SDPA-SqueezeNet+DCNN scheme was compared against state-of-the-art classifiers like Fusion and normalization features based RNN-LSTM (F-RNN-LSTM) [28] and Multi-Class Classification method of Lung Disease using CNN (MCCLLD-CNN) [29], as well as traditional classifiers, including SqueezeNet, LSTM, LinkNet, SVM, and CNN. SqueezeNet is one of the deep neural networks used for image classification. LinkNet is a lightweight neural network architecture designed for semantic segmentation tasks. Long Short-Term Memory (LSTM) is a powerful type of RNN designed to handle sequential data with long-term dependencies. SVM can be used for both classification and regression tasks. Convolutional Neural Network (CNN) is a type of deep learning algorithm specifically designed for image recognition and processing tasks. Additionally, the SDPA-SqueezeNet+DCNN and conventional methodologies were analyzed using the HRCTCov19 dataset.

Lung disease classification is done using FNR, FPR, MCC, F-measure, accuracy, sensitivity, precision, specificity, and NPV evaluation measures.

#### Accuracy

Accuracy is defined as a series of measurements and their real results. Eq (23) defines the mathematical equation of accuracy in which *TP* represents true positive, *TN* represents true negative, *FP* denotes false positive, and *FN* denotes false negative.


$$\text{Accuracy}=\frac{TN+TP}{FP+FN+TP+TN}$$ (23)

#### Precision

A precision metric is the proportion of correctly identified cases. It is detailed in Eq (24).


$$\text{Precision}=\frac{TP}{FP+TP}$$ (24)

#### Sensitivity

The probability of a positive test result is described by sensitivity, which solely considers positive true values. Sensitivity can be expressed using Eq (25).


$$\text{Sensitivity}=\frac{TP}{FN+TP}\times 100$$ (25)

#### Specificity

The chance of exclusively genuine negative values is one way to gauge specificity. The specificity is given by Eq (26).


$$\text{Specificity}=\frac{TN}{FP+TN}\times 100$$ (26)

#### FNR

An alternative word for the FNR is the "miss rate," or false negative rate. It’s defined as the possibility that a real positive will go unnoticed by the test. It is explained in Eq (27).


FNR=FN(FN+TP) (27)

#### FPR

Regardless of whether a test is based on a machine learning model, its false positive rate (FPR) can be used to assess its accuracy. The FPR is described in Eq (28).


$$\text{FPR}=\frac{FP}{FP+TN}$$ (28)

#### F-measure

The F-measure is used to assess the accuracy outcomes. It is represented by Eq (29).


F-measure=TP12FP+FN+TP (29)

#### MCC

Using a contingency matrix method called the Matthews correlation coefficient, one can find the Pearson coefficient for the product-moment correlation between the actual and expected data. This stand-in measure is not affected by the problem of unbalanced datasets. Eq (30) is used to depict it.


$$\text{MCC}=\frac{TP.TN-FP.FN}{\sqrt{\left(TP+FP).(FN+TP).(TN+FP).(TN+FN\right)}}$$ (30)

#### NPV

Negative predictive value is represented by Eq (31).


$$\text{NPV}=\frac{TN}{TN+FN}$$ (31)

### 4.5 Assessment of positive metrics

**Fig 7:**
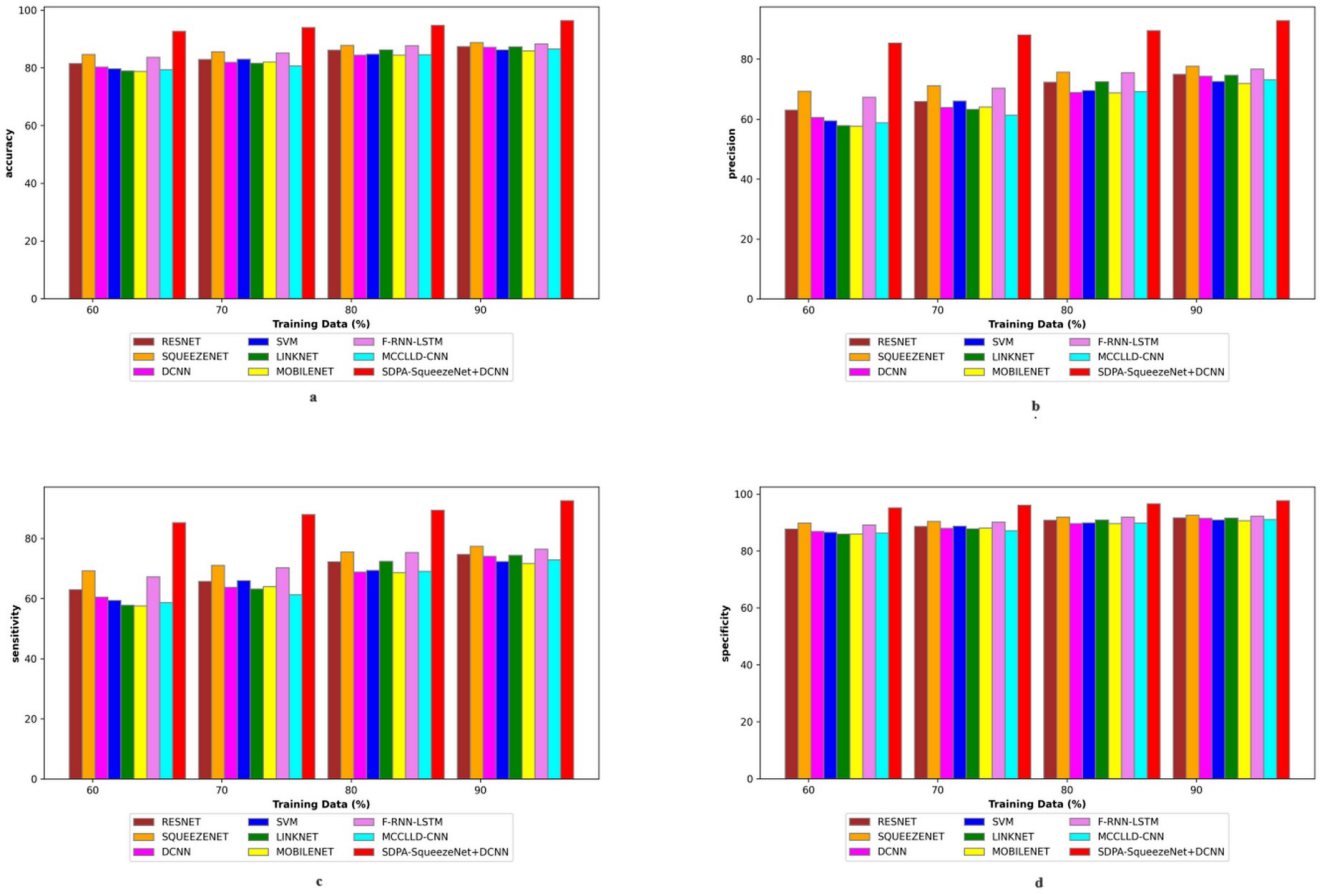
Positive metric analysis on SDPA-SqueezeNet+DCNN and conventional methods.

Fig 7 presents a comparative analysis of the positive metric assessment of the SDPA-SqueezeNet+DCNN model against a range of established techniques, including ResNet, SqueezeNet, LSTM, LinkNet, SVM, CNN, F-RNN-LSTM [28], and MCCLLD-CNN [29], for the classification of lung disease severity based on CT images. This evaluation is crucial for discerning the efficacy of the SDPA-SqueezeNet+DCNN model in accurately classifying the severity of lung diseases. The objective is to identify the model that achieves high accuracy and exhibits superior performance across various evaluation parameters, thereby ensuring dependable and consistent severity classification outcomes. For the training data of 90%, the SDPA-SqueezeNet+DCNN scheme attains an impressive accuracy of 0.918. In contrast, other models such as ResNet, SqueezeNet, LSTM, LinkNet, SVM, CNN, F-RNN-LSTM [28], and MCCLLD-CNN [29] exhibit lower accuracy ratings, indicating their comparatively inferior performance in this task. Furthermore, when evaluating the training data at 90%, the SDPA-SqueezeNet+DCNN method exhibits a notable sensitivity of 0.880, surpassing that of other models. Specifically, the sensitivity values for ResNet, SqueezeNet, LSTM, LinkNet, SVM, CNN, F-RNN-LSTM [28], and MCCLLD-CNN [29] are 0.7043, 0.713, 0.685, 0.661, 0.697, 0.659, 0.728, and 0.709, respectively.

In further evaluating the positive metrics, it is imperative to explore precision and specificity, providing deeper insights into the effectiveness of the SDPA-SqueezeNet+DCNN approach compared to other models. Moreover, the SDPA-SqueezeNet+DCNN scheme exhibits a precision of 0.867 (training data = 70%), showcasing its superior performance compared to ResNet, SqueezeNet, LSTM, LinkNet, SVM, CNN, F-RNN-LSTM [28], and MCCLLD-CNN [29]. This substantial difference underscores the efficacy of the SDPA-SqueezeNet+DCNN approach in accurately classifying instances of lung disease severity. For the training data at 80%, the SDPA-SqueezeNet+DCNN approach achieved the highest precision of 0.868, outperforming ResNet at 0.643, SqueezeNet at 0.659, LSTM at 0.613, LinkNet at 0.627, SVM at 0.648, CNN at 0.631, F-RNN-LSTM [28] at 0.689, and MCCLLD-CNN [29] at 0.667, respectively. Therefore, the positive metric analysis reveals that the SDPA-SqueezeNet+DCNN approach yields significantly higher lung disease severity classification results than conventional methods. The enhancements made, including preprocessing with Improved Weiner filtering, feature extraction using Modified LDN, and severity classification via a hybrid classifier, have collectively contributed to the superior performance of the SDPA-SqueezeNet+DCNN model. These advancements at various stages of the classification process have resulted in more precise and reliable predictions of lung disease severity, highlighting the effectiveness of the SDPA-SqueezeNet+DCNN method.

### 4.6 Assessment of negative metric

**Fig 8:**
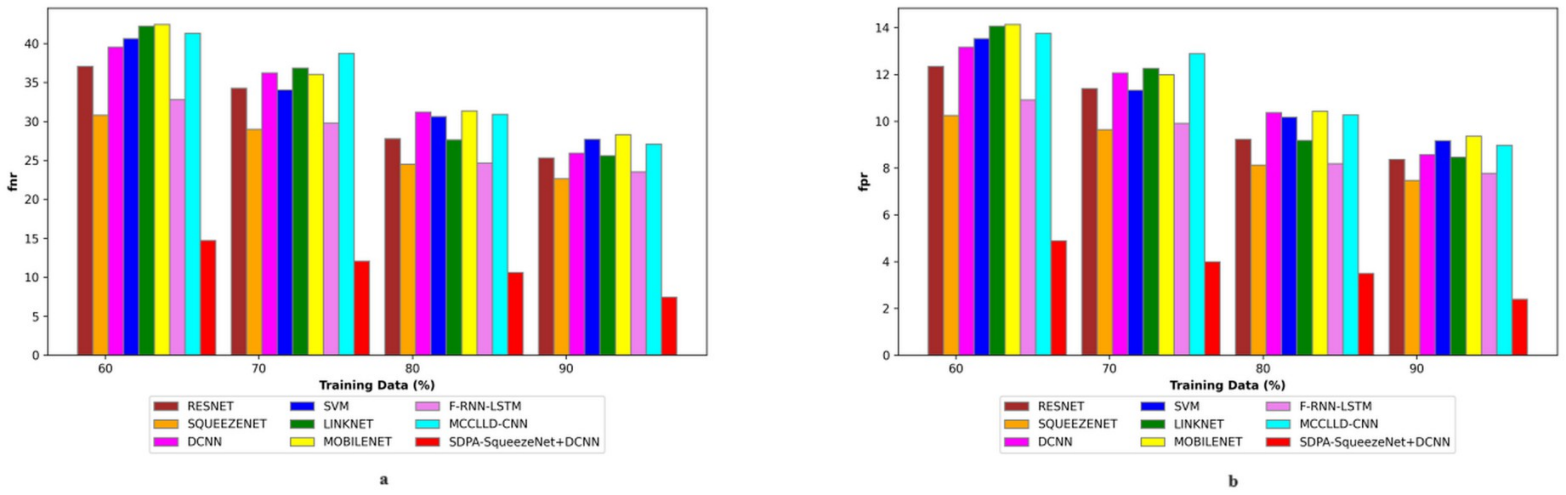
Negative metric analysis on SDPA-SqueezeNet+DCNN and conventional methods.

Fig 8 exposes the comparative analysis of the SDPA-SqueezeNet+DCNN model against ResNet, SqueezeNet, LSTM, LinkNet, SVM, CNN, F-RNN-LSTM [28], and MCCLLD-CNN [29] concerning negative metrics for the classification of lung disease severity using CT images. This evaluation focuses on minimizing negative metric values to ensure the effectiveness of lung disease severity classification. Thus, attaining lower negative metric values is essential for enhancing the overall performance of the classification system. In particular, the FNR of the SDPA-SqueezeNet+DCNN scheme is 12.361 at the training data of 70%, demonstrating the lowest value among all models considered. Conversely, the conventional methods, including ResNet (34.735), SqueezeNet (27.365), LSTM (34.193), LinkNet (33.267), SVM (36.354), CNN (35.175), F-RNN-LSTM [28] (28.592), and MCCLLD-CNN [29] (37.428), exhibit greater FNR values. Furthermore, the FPR of the SDPA-SqueezeNet+DCNN scheme is exceptionally low, ranging from 4.681 to 2.359, while the conventional methodologies exhibit higher FPR values. Thus, the SDPA-SqueezeNet+DCNN model demonstrates its ability to minimize misclassifications and enhance the reliability of the severity classification of lung disease. These findings highlight the robustness of the SDPA-SqueezeNet+DCNN methodology in accurately identifying and classifying lung disease severity.

### 4.7 Assessment of other metric

**Fig 9:**
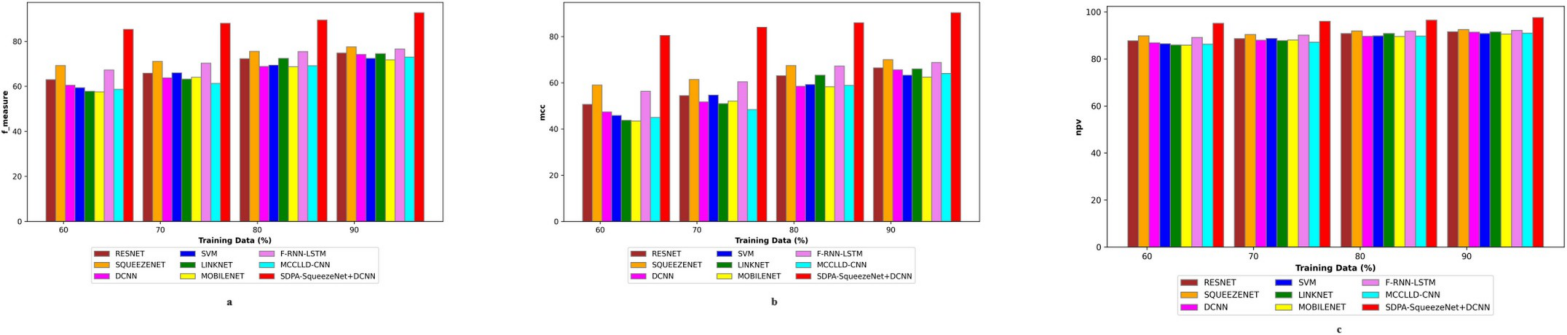
Other metric analysis on SDPA-SqueezeNet+DCNN and conventional methods.

The analysis in Fig 9 presents a comprehensive comparison of various other metrics between the SDPA-SqueezeNet+DCNN model and conventional methodologies, including ResNet, SqueezeNet, LSTM, LinkNet, SVM, CNN, F-RNN-LSTM [28], and MCCLLD-CNN [29], for the classification of lung disease severity using CT images. Maximizing these other metrics is critical for ensuring the accuracy and reliability of lung disease severity classification, as it directly impacts the model’s ability to identify and differentiate between different disease stages properly. For the training data at 90%, the F-measure achieved by the SDPA-SqueezeNet+DCNN approach is 93.259. Meanwhile, traditional approaches maintain lower F-measure values: ResNet = 72.648, SqueezeNet = 74.168, LSTM = 73.152, LinkNet = 70.935, SVM = 72.193, CNN = 67.395, F-RNN-LSTM [28] = 69.241 and MCCLLD-CNN [29] = 67.163, respectively. According to the NPV metric, the SDPA-SqueezeNet+DCNN approach consistently attained the highest NPV compared to the conventional methods across all the training data. Hence, the analysis of other measures reaffirms the superior performance of the SDPA-SqueezeNet+DCNN method in lung disease severity classification. Through the integration of advanced techniques such as Improved Weiner filtering for preprocessing, Modified LDN for feature extraction, and a hybrid classifier, the SDPA-SqueezeNet+DCNN model consistently outperforms traditional methods.

### 4.8 Ablation study on SDPA-SqueezeNet+DCNN

Ablation analysis involves systematically disabling or removing particular components within a system to observe how it affect behavior or performance. By isolating individual elements, this method helps discern their contributions to system functionality, aiding in understanding underlying mechanisms and optimization endeavors. The ablation analysis on the SDPA-SqueezeNet+DCNN model, over the Modified SegNet without Wiener filter, SqueezeNet and DCNN with images directly as input, Images + Wiener filter as input, Images + Wiener filter + segmentation, Model with Wiener filter, Model with Conventional Segnet and Model with MLDN for Lung Disease Segmentation and Severity Classification using CT images, is depicted in Table 5. Furthermore, the specificity of the SDPA-SqueezeNet+DCNN approach is 0.960, surpassing the specificity values of the model with Modified SegNet without Wiener filter, SqueezeNet and DCNN with images directly as input, Images + Wiener filter as input, Images + Wiener filter + segmentation, Model with Wiener filter, Model with Conventional Segnet and Model with MLDN, which obtained the lowest specificity values of 0.9234, 0.9187, 0.923, 0.930, 0.933, 0.936, and 0.931respectively. Additionally, the FPR of the SDPA-SqueezeNet+DCNN approach is 0.039854, demonstrating a lower rate compared to the models with Modified SegNet without Wiener filter (0.076), SqueezeNet and DCNN with images directly as input (0.081), Images + Wiener filter as input (0.076), Images + Wiener filter + segmentation (0.069), Model with Wiener filter (0.066), Model with Conventional Segnet (0.063) and Model with MLDN (0.068) respectively. Hence, the ablation analysis reaffirms the superior performance of the SDPA-SqueezeNet+DCNN method in lung disease severity classification. Through the integration of advanced techniques such as Improved Weiner filtering for preprocessing, Modified LDN for feature extraction, and a hybrid classifier, the SDPA-SqueezeNet+DCNN model consistently performs better in all the diverse metrics.

**Table 5:** Ablation evaluation on SDPA-SqueezeNet+DCNN over modified SegNet without Wiener filter, SqueezeNet and DCNN with images directly as input, Images + Wiener filter as input, Images + Wiener filter + segmentation, model with Wiener filter, model with conventional Segnet and model with MLDN.

Metrics	Modified SegNet without Wiener filter	SqueezeNet and DCNN with images directly as input	Images + Wiener filter as input	Images + Wiener filter + segmentation	Model with Wiener filter	Model with Conventional Segnet	Model with MLDN	Proposed (SDPA-SqueezeNet+DCNN) model
**Accuracy**	0.88496	0.877988	0.88446	0.895667	0.900647	0.904631	0.897659	0.93999
**Sensitivity**	0.76965	0.755721	0.76865	0.791045	0.800995	0.808955	0.795025	0.879602
**Specificity**	0.92344	0.918798	0.92311	0.930588	0.933909	0.936566	0.931916	0.960146
**Precision**	0.77041	0.756474	0.76942	0.791833	0.801793	0.809761	0.795817	0.880478
**F_measure**	0.77003	0.756098	0.76903	0.791439	0.801394	0.809358	0.795421	0.88004
**MCC**	0.69332	0.674743	0.69200	0.721872	0.735148	0.745769	0.727182	0.840027
**NPV**	0.92314	0.918493	0.92280	0.930279	0.933599	0.936255	0.931607	0.959827
**FPR**	0.07655	0.081202	0.07688	0.069412	0.066091	0.063434	0.068084	0.039854
**FNR**	0.23034	0.244279	0.23134	0.208955	0.199005	0.191045	0.204975	0.120398

### 4.9 Statistical evaluation of accuracy

Statistical analysis entails evaluating numerous iterations of diverse models, each iteration executed multiple times. Throughout this procedure, analysts compute statistical metrics such as mean, median, maximum, minimum, and standard deviation, applying them once to the results of these runs. Table 6 describes the statistical assessment of the SDPA-SqueezeNet+DCNN model, contrasting it with ResNet, SqueezeNet, LSTM, LinkNet, SVM, CNN, F-RNN-LSTM [28], and MCCLLD-CNN [29] for Lung Disease Segmentation and Severity Classification using CT images. Considering the minimum statistical metric, the accuracy achieved by the SDPA-SqueezeNet+DCNN scheme is 0.927, markedly higher than the accuracy ratings of traditional methods, precisely, ResNet = 0.814, SqueezeNet = 0.846, LSTM = 0.803, LinkNet = 0.797, SVM = 0.789, CNN = 0.788, F-RNN-LSTM [28] = 0.836 and MCCLLD-CNN [29] = 0.794, respectively. Furthermore, the SDPA-SqueezeNet+DCNN method accomplished the maximum accuracy score of 0.944 for the mean statistical metric, while the conventional strategies recorded the lowest accuracy ratings.

**Table 6:** Statistical analysis of accuracy.

Methods	Minimum	Standard Deviation	Mean	Maximum	Median
**SqueezeNet**	0.846	0.017	0.867	0.887	0.867
**ResNet**	0.814	0.023	0.844	0.873	0.845
**LSTM**	0.803	0.026	0.834	0.871	0.832
**LinkNet**	0.797	0.024	0.834	0.862	0.839
**SVM**	0.789	0.034	0.835	0.872	0.839
**CNN**	0.788	0.027	0.828	0.859	0.832
**F-RNN-LSTM [28]**	0.836	0.019	0.862	0.883	0.864
**MCCLLD-CNN [29]**	0.794	0.029	0.828	0.865	0.826
**SDPA-SqueezeNet+DCNN**	0.927	0.013	0.944	0.963	0.944

### 4.10 Analysis of confusion matrix

**Fig 10:**
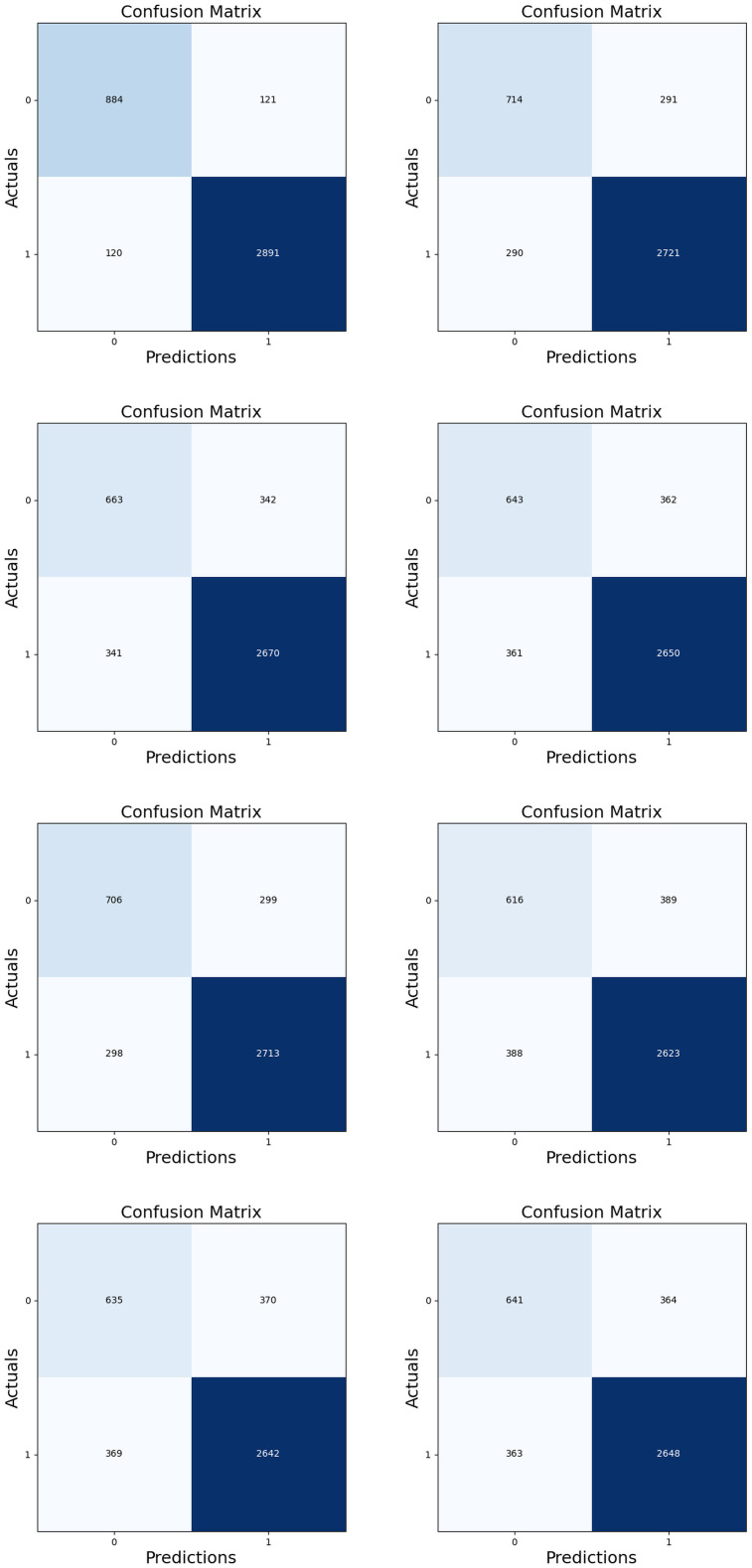
Analysis on Confusion Matrix a) F-RNN-LSTM [28] b) MCCLLD-CNN [29] c) CNN d) LinkNet e) LSTM f) SqueezeNet g) SVM h) ResNet and i) SDPA-SqueezeNet+DCNN.

A confusion matrix serves as a tabular representation commonly employed to assess the effectiveness of a classification model. It condenses the model’s predictions regarding a dataset with known true values. The confusion matrix is structured as a grid with rows and columns representing the actual and predicted classes. In this layout, each row signifies the actual class, while each column signifies the predicted class. Fig 10 presents the confusion matrix depicting the performance of both the SDPA-SqueezeNet+DCNN method and conventional approaches (F-RNN-LSTM [28], MCCLLD-CNN [29], CNN, ResNet, LinkNet, LSTM, SqueezeNet, and SVM). In this matrix, 0 indicates True, while 1 represents False. Moreover, the TPR achieved using the SDPA-SqueezeNet+DCNN approach is 884, significantly exceeding that of conventional strategies: F-RNN-LSTM [28] = 706, MCCLLD-CNN [29] = 616, CNN = 643, ResNet = 645, LinkNet = 663, LSTM = 641, SqueezeNet = 714, and SVM = 635. Additionally, the SDPA-SqueezeNet+DCNN scheme records the lowest FNR at 120, while F-RNN-LSTM [28], MCCLLD-CNN [29], CNN, ResNet, LinkNet, LSTM, SqueezeNet, and SVM exhibit higher FNR scores. Consequently, the confusion matrix analysis reveals that the SDPA-SqueezeNet+DCNN method achieved better-predicted values than conventional methods. Specifically, the SDPA-SqueezeNet+DCNN approach demonstrated superior outcomes for lung disease severity classification, as evidenced by its ability to identify true positive cases precisely and minimize false pessimistic predictions.

## 5 Conclusion

This paper introduced a new framework called Modified Segnet-based Lung Disease Segmentation and Severity Classification (MSLDSSC). The MSLDSSC model consists of four phases: preprocessing, Segmentation, feature extraction, and classification. Initially, the input image underwent preprocessing using an improved Wiener filter technique. This technique estimates the power spectral density of the original and noisy images and computes the signal-to-noise ratio (SNR) assisted by the Peak Signal-to-Noise Ratio (PSNR) to evaluate image quality. Next, the preprocessed image underwent Segmentation to identify and separate the Region of Interest (RoI) from the background objects in the lung image. We employed a Modified Segnet mechanism with a proposed hard tanh-Softplus activation function for effective Segmentation. Features such as Modified Local Directional Number Pattern (MLDN), entropy with Median Robust Extended Local Binary Pattern (MRELBP), shape features, and deep features were extracted following Segmentation. After the feature extraction phase, the retrieved feature set was input into a hybrid severity classification model. This hybrid model comprised two classifiers: SDPA-Squeezenet (Scale Dot Product Attention-based Squeezenet) and DCNN. These classifiers train on the retrieved feature set and effectively classify the severity level of lung diseases. For the training data at 80%, the SDPA-SqueezeNet+DCNN approach achieved the highest precision of 0.868, outperforming ResNet at 0.643, SqueezeNet at 0.659, LSTM at 0.613, LinkNet at 0.627, SVM at 0.648, CNN at 0.631, F-RNN-LSTM [28] at 0.689, and MCCLLD-CNN [29] at 0.667, respectively. The proposed deep learning models (M-Segnet and Hybrid Squeezenet-CNN) achieve impressive classification accuracy for lung diseases. Thus, the M-Segnet and Hybrid Squeezenet-CNN architectures offer accurate, automated, and efficient lung disease classification, benefiting both patients and healthcare providers. However, Deep learning models, including M-Segnet and Squeezenet-CNN, can struggle with generalizing well to unseen data. If the training dataset lacks diversity or contains biases, the model may not perform optimally on real-world CT images from different sources or patient populations. Future work will be extended by optimizing the hyperparameters, adjusting architectures, and incorporating novel techniques into the M-Segnet and Hybrid Squeezenet-CNN. This will explore more efficient and lightweight architectures to improve inference speed without compromising accuracy. Moreover, the development of fusion techniques will be leveraging complementary features from different modalities like CT, X-ray, and MRI for comprehensive lung disease diagnosis.
